# Supplementary material for: Incorporating Contact Network Structure in Cluster Randomized Trials
Source: Sci Rep. 2015 Dec 3;5:17581. doi: 10.1038/srep17581 (PMC4668393; doi:10.1038/srep17581)
Supplement: Supplementary Information [file srep17581-s1.pdf]

Incorporating Contact Network Structure in Cluster Randomized Trials

Supplementary Material

Patrick C. Staples*[1], Elizabeth L. Ogburn†[2], and Jukka-Pekka Onnela‡[1]

[1]Department of Biostatistics, Harvard University, Boston, MA 02115, USA
[2]Department of Biostatistics, Johns Hopkins University, Baltimore MD, 21205, USA

*email: patrickstaples@fas.harvard.edu
†email: eogburn@jhsph.edu
‡email: onnela@hsph.harvard.edu

In this supplement, we provide additional details for a few topics discussed in the main paper. Section S1 demonstrates a simple approach to modeling infectious spread with between-cluster mixing using ordinary differential equations, and compares this result to the simulation approach introduced in the paper. Section S2 describes the stochastic blockmodel and provides details for the specific model we used in our paper. Section S3 connects our definition of between-mixing parameter γ with a common metric used in applications of network science. Section S4 describes how the Intracluster Correlation Coefficient is defined, and we show estimates of this quantity for our simulations. Finally, Section S5 shows the degree distribution for the empirical cell phone network, with discussion.

S1: Ordinary Differential Equation approach to epidemic spreading with between-cluster mixing.

One of the most common approaches to investigating the spread of an epidemic on networks is Ordinary Differential Equations (ODEs) [1,2]. ODEs are functions of a variable in terms of its derivatives. Compartmental models for epidemic spread can use ODEs to specify the rate of change for individuals in terms of others. A common assumption used to specify ODEs for epidemic spread is *mass action*, in which the spread of an infection depends only on the proportion of individuals in each compartment. For example, an SI compartmental model assumes that individual i is either infected ($I_i(t) = 1$) or not infected but susceptible ($S_i(t) = 1$) at any time t. These two statuses are mutually exclusive, and $S_i(t) = 1 - I_i(t)$. An ordinary differential equation that assumes mass action would specify the change in the total proportion of infected individuals $I(t) := \langle I_i(t)\rangle$ in terms of the infected proportion $I(t)$ at time t. If we assume mass action, we may model the rate of infectious growth in an SI compartmental model as proportional to the proportion of infected individuals multiplied by the proportion of susceptible individuals:

$$\frac{dI(t)}{dt} = pS(t)I(t) = p(1 - I(t))I(t) \tag{1}$$

In this paper, we consider a collection of $c = 1, ..., C$ cluster pairs, with one cluster in each pair assigned to the treatment condition $r = 1$ and the other to control $r = 0$. Furthermore, we assume that clusters are mixed according to mixing parameter γ, For the SI compartmental model, $I_{irc}(t) = 1$ if individual i is infected and 0 otherwise. We may assume that the spread of an infection across the network pair is a mass action ODE as above, with a simple modification. Let $I_{rc}(t) = \langle I_{irc}(t)\rangle$ represent the proportion of infected nodes in cluster pair c at discrete time t. Individual i may contact an individual j in the opposing cluster with probability γ. In this case, the probability of a successful infection requires that i is suspectible and j is infectious. Mass action dictates that the rate of change for each cluster depends only on the proportion of individuals in each infectious status for either cluster, which is now sum of ODEs weighted by mixing parameter γ:

$$\frac{\partial I_{0c}(t)}{\partial t} = [(1-\gamma)I_{0c}(t)p_0 + \gamma I_{1c}(t)p_1]\,(1 - I_{0c}(t)) \tag{2}$$

$$\frac{\partial I_{1c}(t)}{\partial t} = [(1-\gamma)I_{1c}(t)p_1 + \gamma I_{0c}(t)p_0]\,(1 - I_{1c}(t)) \tag{3}$$

According to Supplementary Equations 2 and 3, if $\gamma = 0$, the rate of infection in each cluster is identical to Supplementary Equation 1. As γ approaches 1/2, the difference in the proportion of infected individuals in the two treatment arms decreases to no difference.

The ODE approach is quite comparable to the stochastic approach we chose for the paper. To show this, we created network clusters with every node connected to each other in the cluster, performed degree-corrected rewiring, simulated an infectious processes with unit infectivity on the pair according to the paper, and averaged the proportion of infections at each time step. Supplementary Figure 1 shows the infection rates over time for a range of mixing values $\gamma = \{0.0, 0.1, 0.2, 1\}$. The solid lines shows the average of the network simulations. The dashed lines show the a numerical solution to Supplementary Equations 2 and 3. The two are comparable, suggesting that differential equations and network simulations can approximately interchangeably describe the same infectious process.

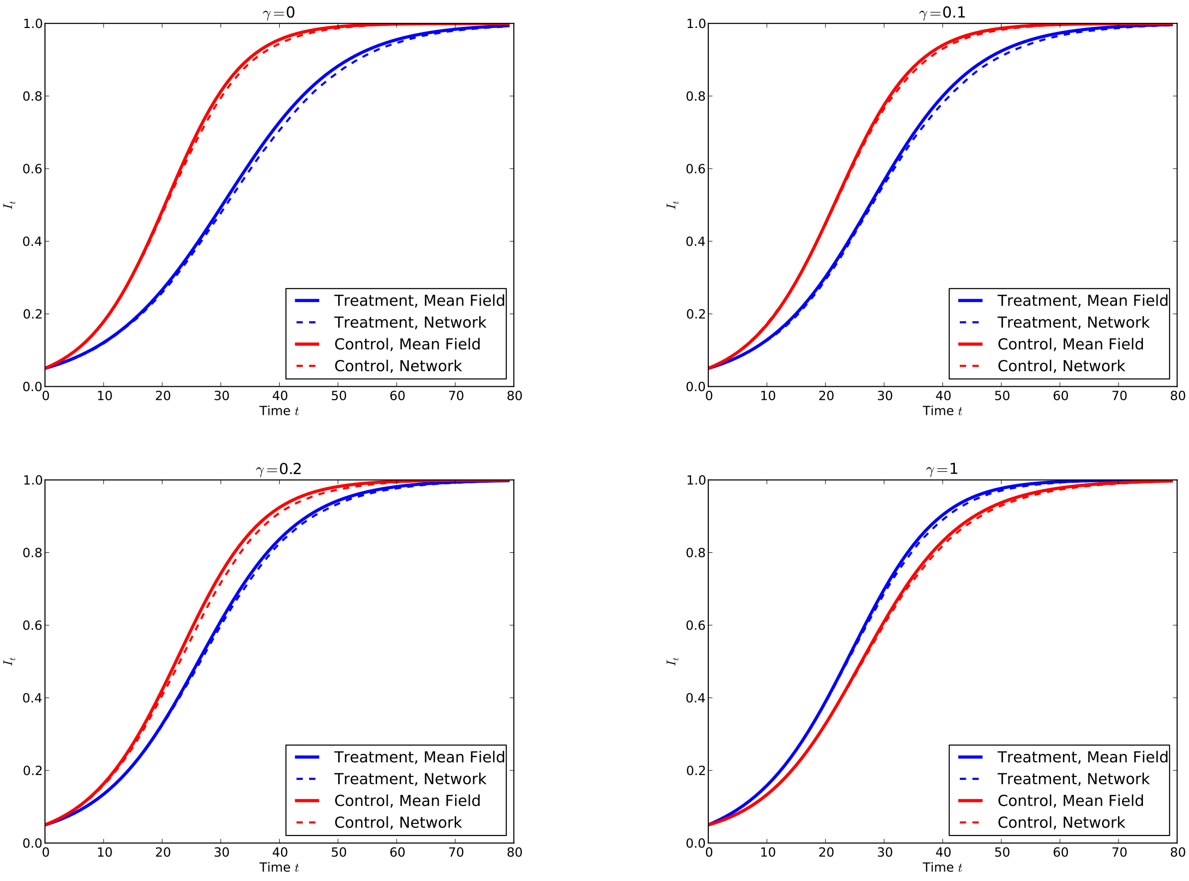

Supplementary Figure 1: The proportion of infections over time. The solid line is the mass action rate equation, and the dashed lines are the mean of simulations of an infectious process on a complete (fully-connected) network. The infectious process was simulated for $\gamma = \{0.0, 0.1, 0.2, 1\}$, matching Figure 5. As γ approaches $1/2$, the difference in infection rates in two clusters in a pair decreases, demonstrated by the red and blue curves approaching each other. When $\gamma = 1$, the relative rates of infections switch.

Where the differential equation approach assumes individuals contact everyone in the population, infections spreading through fixed networks only allow contact through existing edges. This *redundant contact effect*[3] causes infections through networks to be slightly slower, also observable in Supplementary Figure 1.

S2: Modularity and Between-Mixing Parameter γ

Our definition of between-mixing parameter γ (Equation 2) has a convenient interpretation in terms of findings in network science. Modularity Q is a measure of how well the individuals in a network and their relationships fit into mutually exclusive groups[4]. For CRTs, we assume the natural groupings to be the two treatment arms. If $Q = 1$, all edges exist within treatment arms. If $Q = -1$, all edges are between the two treatment arms. The definition of modularity is written in the same terms as γ:

$$Q := \frac{1}{2m} \sum_{ij} \left(A_{ij} - \frac{k_i k_j}{2m} \right) \delta(r_i, r_j) \tag{4}$$

If the individuals between the two treatment arms have equal numbers of edges, $\sum_{ij} \frac{k_i k_j}{(2m)^2} \delta(r_i, r_j) = 1/2$, and $\gamma = 1/2 - Q$. Therefore, if modularity can be computed, so can the mixing between the two treatment arms. More generally, γ is entirely a function of cluster structure matrix $\mathbf{A}$ and treatment assignments, so if an experimenter

knows the structure of relationships among individuals in the study, they may calculate the estimate the amount of mixing between the two treatment arms.

S3: Details on the Stochastic Blockmodel

A stochastic blockmodel (SBM) is a probablistic network model, which means that the probability of an edge existing between nodes i and j is specified by probability $p_{i,j}$. SBM assumes that each network node is a member of a exactly one block in a partition of b blocks $\mathcal{B} = B_1, ..., B_b$, and the probability $p_{i,j}$ of a connection between nodes i and j depends only on each node's block membership. Denote the block membership of node i as B_i. A probability matrix $P_{b\times b}$ describes all edge probabilities for a network, with $p_{i,j} = P_{B_i,B_j}$.

In our study, we imitated within-cluster community structure using a SBM. We assume each cluster is comprised of blocks arranged in a triangular lattice structure. Blocks of nodes may be thought of near each other in geographic location, and while most edges are contained within each block, blocks share a few edges according to a triangular spatial pattern. We organized clusters into 10 equally-sized blocks, and individuals within each block are connected to others within their block such that average within-block degree is $\frac{9}{10}\langle k\rangle$. For between-block connections, we also assume that each edge between members of blocks share a total between-block degree of $\frac{1}{10}\langle k\rangle$ with adjacent blocks according to the lattice structure, and no edges with all other blocks. A diagram of this network ensemble is shown in Supplementary Figure 2.

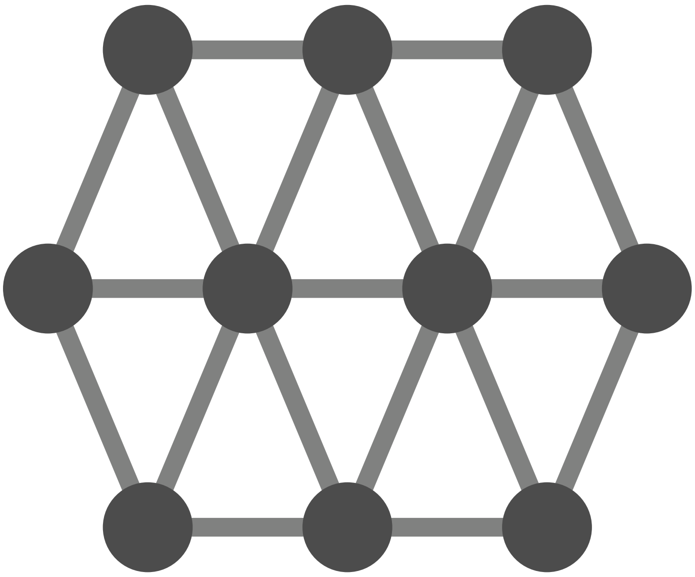

Supplementary Figure 2: 10 communities or blocks within clusters were created according to the stochastic blockmodel, with a small probability of community ties in a triangular lattice. Edge probabilities were selected to preserve the average degree of a random network.

S4: The ICC

The Intracluster Correlation Coefficient (ICC) is a measure of the average correlation between individual outcomes within a cluster. The ICC assumes that the correlation is identical for all pairs of individuals within a cluster, and is constant across clusters. The ICC can also be expressed as the ratio of between-cluster variance to the total outcome variance in the study[5]. In the case of binary outcomes, this value may be expressed as[6]

$$\text{ICC} = \frac{\langle \pi_c(1-\pi_c)\rangle}{\langle \pi_c\rangle(1-\langle \pi_c\rangle)} \tag{5}$$

where π_c is the proportion of infections in cluster c and $\langle \cdot \rangle$ is the average over all clusters in a trial. We calculated the ICC this value for each network ensemble and value of γ in our simulations. These results are shown in Supplementary Figure 3.

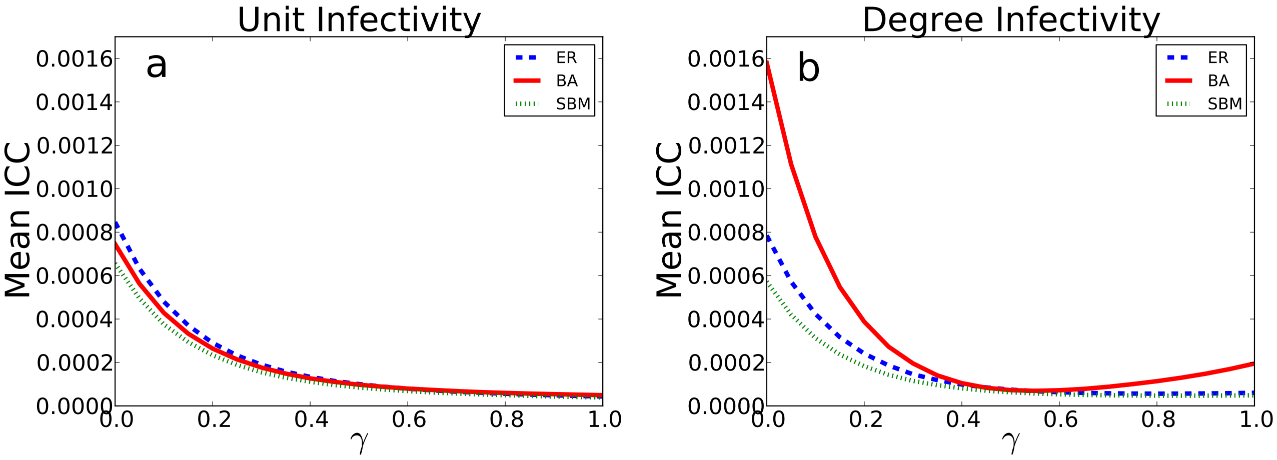

Supplementary Figure 3: ICCs from Scenario 1, averaged over all simulations. ICC values are shown for unit infectivity (Panel a) and degree infectivity (Panel b), as well as each within-cluster structure and extent of between-cluster mixing specified in our simulations.

These values are quite low, but not very far from typical values[7] and lower values have been reported in actual trials[6]. These values for the ICC are low because in our design, the data is collected for each cluster pair when the average proportion of infections within each pair is 10%, which results in relatively low variation in infection proportions for each cluster.

Like power, the relative value of the ICC depends on within-cluster structure, the amount of between-cluster mixing, and infectivity. In the case of unit infectivity, the ICC shrinks as between-cluster mixing increases for all within-cluster structures. However, in many power calculation formulas[8], lower values of ICC indicate increased power, not less. This shows that even if sample size calculations account for within-cluster correlations as measured by the ICC, power can be reduced by other trial features, such as the extent of between-cluster mixing.

S5: Degree Distribution for an Empirical Cell Phone Network

The main paper specifies two definitions for an edge between callers in the cell phone network, which are, respectively, unweighted or weighted by the number of total number of calls made between each pair of callers. The empirical degree distribution for both definitions are found in Supplementary Figure 4.

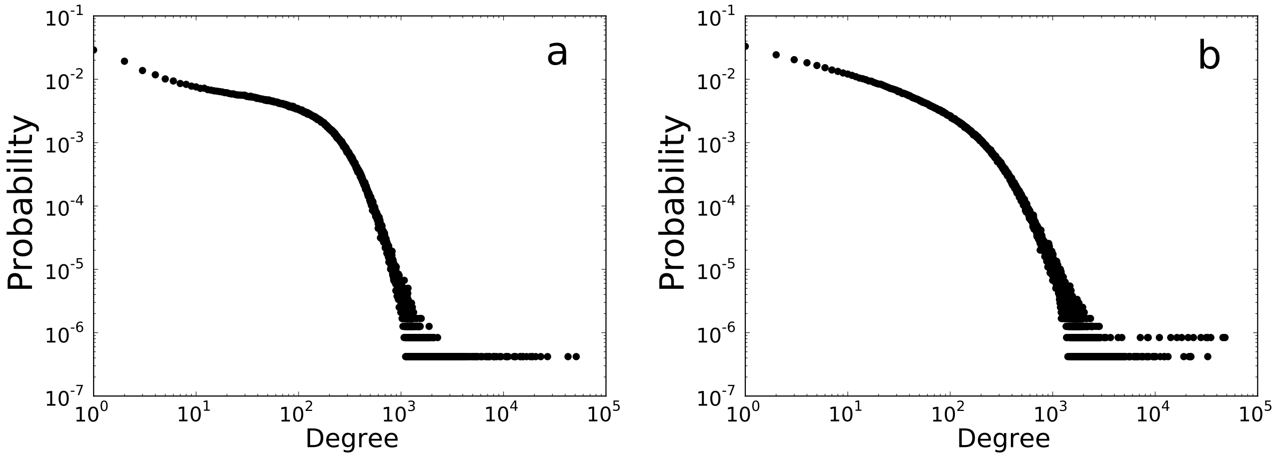

Supplementary Figure 4: The empirical degree distribution for the calling network dataset. Panel a corresponds to Definition 1 (unweighted), and Panel b corresponds to Definition 2 (weighted).

Focusing on Panel a, we notice three distinct regimes. The vast majority of callers make calls with $1-100$ others. The distribution of those who call a large number $(100-1000)$ of others follows a nearly straight line on these log-log plots, which is indicative of a power-law for this segment. Finally, a few singular callers are found to call a very large number (>1000) of callers within the quarter. The general shape is similar for both the unweighted and weighted definitions. This degree distribution is in accordance to similar datasets analyzed in the literature [9].

References

[1] Roy M Anderson and Robert McCredie May. *Infectious diseases of humans*, volume 1. Oxford university press Oxford, 1991.

[2] Romualdo Pastor-Satorras, Claudio Castellano, Piet Van Mieghem, and Alessandro Vespignani. Epidemic processes in complex networks. *arXiv preprint arXiv:1408.2701*, 2014.

[3] Tao Zhou, Jian-Guo Liu, Wen-Jie Bai, Guanrong Chen, and Bing-Hong Wang. Behaviors of susceptible-infected epidemics on scale-free networks with identical infectivity. *Physical Review E*, 74(5):056109, 2006.

[4] Brian Karrer and M. E. J. Newman. Stochastic blockmodels and community structure in networks. *Phys. Rev. E*, 83:016107, Jan 2011.

[5] Sally M Kerry and J Martin Bland. The intracluster correlation coefficient in cluster randomisation. *Bmj*, 316(7142):1455–1460, 1998.

[6] Sandra Eldridge and Sally Kerry. *A practical guide to cluster randomised trials in health services research*, volume 120. John Wiley & Sons, 2012.

[7] Rebecca M Turner, Simon G Thompson, and David J Spiegelhalter. Prior distributions for the intracluster correlation coefficient, based on multiple previous estimates, and their application in cluster randomized trials. *Clinical Trials*, 2(2):108–118, 2005.

[8] RJ Hayes and S Bennett. Simple sample size calculation for cluster-randomized trials. *International journal of epidemiology*, 28(2):319–326, 1999.

[9] Jukka-Pekka Onnela, Samuel Arbesman, Marta C González, Albert-László Barabási, and Nicholas A Christakis. Geographic constraints on social network groups. *PLoS one*, 6(4):e16939, 2011.
